# Evaluating malaria burden in children under-five and intervention outcomes in Tarkwa-Nsuaem municipality

**DOI:** 10.1186/s12879-025-10705-z

**Published:** 2025-02-28

**Authors:** Anafo Abdulzeid, Senyefia Bosson-Amedenu, Vincent Uwumboriyhie Gmayinaam, Appiah Enock, Selasi Ocloo, Joseph Acquah

**Affiliations:** 1 Department of Mathematical Sciences, University of Mines and Technology, P.O. Box 237, Tarkwa, Ghana; 2 Department of Mathematics, Statistics and Actuarial Science, Takoradi Technical University, Takoradi, Ghana; 3 Department of Biostatics and Epidemiology, University of Health and Allied Science, Hohoe, Ghana; 4 Department of Engineering, Ashesi University, Brekuso, Ghana

**Keywords:** Malaria, ITN coverage, Vector control, Case management, Incidence rate, Mortality rate, Case fatality rate, Severe malaria, Time-series analysis

## Abstract

**Background:**

Malaria is a significant public health burden, particularly in sub-Saharan Africa. Despite global efforts to reduce malaria incidence, various challenges, including socio-economic disparities, insecticide resistance, and climatic factors, continue to hamper malaria elimination in Ghana. Over the years, several interventions have been implemented to combat malaria. However, the implementation of these malaria interventions and their association with the malaria burden remains unclear.

**Aim:**

This study evaluated the epidemiological behaviour of malaria in Tarkwa-Nsuaem Municipality from 2013 to 2023.

**Materials and methods:**

Malaria incidence, severe cases, and mortality among children under five from 2013 to 2023 were obtained from the Tarkwa-Nsuaem Health Directorate. Meteorological data were sourced from the Global Climate Monitor, while intervention coverage data were extracted from the Malaria Atlas Project. The study employed the Mann-Kendall test to assess trends and applied Joinpoint regression to detect significant shifts in malaria incidence, severe cases, and mortality. Additionally, data on insecticide-treated net coverage and case management treatment were analyzed to evaluate intervention effectiveness. To further assess the influence of climate factors on malaria incidence, a Seasonal AutoRegressive Integrated Moving Average with Exogenous Variables model was applied. The best-fitting model, SARIMAX(1,1,1)x(1,1,1,12), incorporated rainfall and temperature as exogenous predictors to capture the temporal dynamics and seasonal variations in malaria incidence.

**Results:**

Over the study period, 110,737 malaria cases were reported, with an annual mean incidence rate of 242.37 cases per 1,000 population. Malaria incidence increased significantly by 12.48% from 109.63 cases per 1,000 in 2013 to 234.41 in 2023 (*p* = 0.02). ITN coverage fluctuated between 27.21% and 51.82%, and treatment coverage improved steadily to 62.08%. Malaria-related deaths decreased significantly, with zero deaths reported since 2020. However, severe malaria cases showed a fluctuating trend, decreasing by 80.6% from 2013 to 2018, followed by a 110.3% increase from 2018 to 2023. The AutoRegressive Integrated Moving Average with Exogenous Variables model results indicated that rainfall was a significant predictor of malaria incidence (*p* = 0.032), while temperature did not show a statistically significant impact (*p* = 0.927). The model successfully captured historical trends and seasonal variations.

**Conclusions:**

The study showed a significant reduction in malaria-related mortality in Tarkwa-Nsuaem, likely attributable to improved case management and treatment coverage. However, the fluctuating ITN coverage and the recent rise in severe cases warrant further investigation. Targeted interventions, especially in mining areas, and more consistent vector control measures are needed to sustain progress and further reduce malaria incidence.

## Background

Malaria is a significant global public health challenge, with a high burden of disease primarily affecting low- and middle-income countries in sub-Saharan Africa, South Asia, and Latin America [1–3]. According to the World Health Organization [4], there were an estimated 247 million cases of malaria worldwide in 2021, with an estimated 619,000 deaths compared to compared to 625,000 in 2020 [4]. Despite the burden of malaria on the world, recent years have seen advances in lowering its incidence. The incidence of malaria decreased by 27% globally between 2010 and 2019, while the number of fatalities attributable to malaria decreased by 60% according to WHO [4]. Malaria elimination efforts have been hampered by the COVID-19 epidemic, increasing concerns that gains may be reversed [5, 6]. During the COVID-19 pandemic between 2020 and 2021, there were an additional 13 million malaria cases and 63,000 more malaria deaths [4].

The history of malaria control efforts in Ghana dates back to the 1950s when drain construction, chloroquine impregnated salts, aerial spraying and weekly swallowing of Daraprim called “Sunday-Sunday as a malaria preventive strategy [7, 8]. Since then, several interventions have been introduced to control malaria in Ghana. In the early 2000s, the distribution of insecticide treated nets (ITNs) began as a strategy for malaria control in Ghana. This intervention was scaled up in 2003 with the launch of the Ghana National Malaria Control Program (NMCP) and the distribution of long-lasting insecticide-treated nets (LLINs) as part of the Roll Back Malaria (RBM) initiative [9]. In 2004, the use of Artemisinin-based Combination Therapy (ACT) for the treatment of uncomplicated malaria was introduced as the first-line treatment in Ghana, replacing chloroquine and sulfadoxine-pyrimethamine (SP) [10]. Intermittent preventive treatment in pregnancy (IPTp) was also introduced in 2005 as part of Ghana’s strategy for the prevention of malaria in pregnant women [11]. This intervention involves the administration of SP to pregnant women during antenatal care visits. SMC, a preventive intervention for children aged 3 to 59 months, was first implemented in Ghana in 2015 in selected districts with high malaria burden [12]. The RTS, S malaria vaccine was introduced in Ghana in 2019 as part of a pilot program in selected districts across the country. The vaccine is administered to children aged 5 to 17 months as a complement to existing malaria control interventions. Multiple studies have examined malaria trends and interventions across Ghana and other African countries. Aregawi et al. analyzed malaria morbidity and mortality trends in Ghana and reported a decline in malaria-related hospitalizations following the introduction of major interventions [13]. Similarly, a study by Owusu et al. highlighted the effectiveness of ITN in reducing malaria transmission [14], while Adjei et al. explored the socio-economic determinants of malaria prevalence [15, 16]. Outside Ghana, studies from Nigeria, Kenya, and Uganda have reported similar trends [17], showing a decline in malaria incidence following intervention programs but highlighting persistent challenges such as drug resistance, changing mosquito behavior, and variations in intervention coverage [18].

The relationship between the implementation and effectiveness of malaria prevention strategies and epidemiological trends over the past decade remains unclear, necessitating further research and evaluation across diverse settings. Tarkwa-Nsuaem Municipality, a major mining hub in Ghana’s Western Region, presents a unique case for such an investigation. Despite the implementation of malaria control interventions, such as the distribution of ITN and the provision of treatment, malaria cases significantly increased, with the incidence rate rising from 109.63 per 1,000 population in 2013 to 234.41 per 1,000 in 2023. The municipality also presents unique challenges for malaria control due to the environmental and socio-economic factors associated with mining activities. Mining operations create water bodies that serve as breeding grounds for malaria-transmitting mosquitoes, while the influx of migrant workers increases population mobility, contributing to malaria transmission. It is with this, therefore, that this study examined the epidemiological behaviour and trends of malaria in Tarkwa-Nsuaem from 2013 to 2023 and assessed changes in the coverage of major interventions and their relationship with malaria burden.

## Methods

### Study design, setting and population

**Fig. 1:**
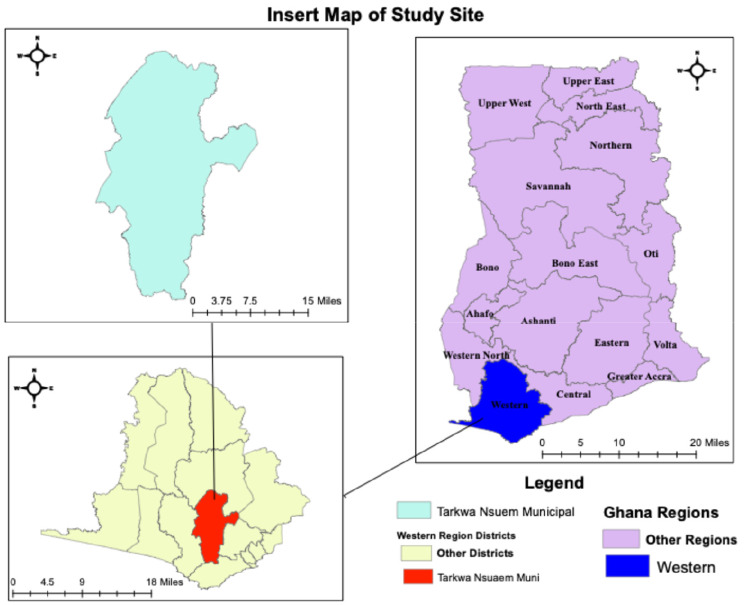
A Map of Tarkwa Nsuaem Municipal

A time-series study was conducted in Tarwa-Nsuaem municipality from 2013 to 2023. The Tarkwa-Nsuaem Municipal District is situated between Latitude 4°N and 5°40’N and Longitudes 10°45’W and 20°10’W. It shares its boundaries with the following districts: to the north, the Wassa Amenfi East District; to the south, the Ahanta West District; to the west, the Nzema East Municipal; and to the east, the Mpohor Wassa East. The total land area of the municipality is approximately 2354 square kilometres [19]. Situated in the South-Western Equatorial Climate Zone is the Municipality. August and March have temperatures ranging from 26 to 30 degrees Celsius. Most of the year has an average of seven hours of sunshine every day. Throughout the year, relative humidity is often high, ranging from 70 to 80 per cent during the dry season to 75 to 78 per cent during the wet season. In Ghana, the Municipality receives the most rainfall. With a double maximum rainfall occurring from March to September, the primary rainy season, it has an annual mean rainfall of 1,500 mm. The amount of rainfall in a given area has a significant impact on the density and distribution of vector populations [19]. (Fig. 1)

### Data collection

**Fig. 2:**
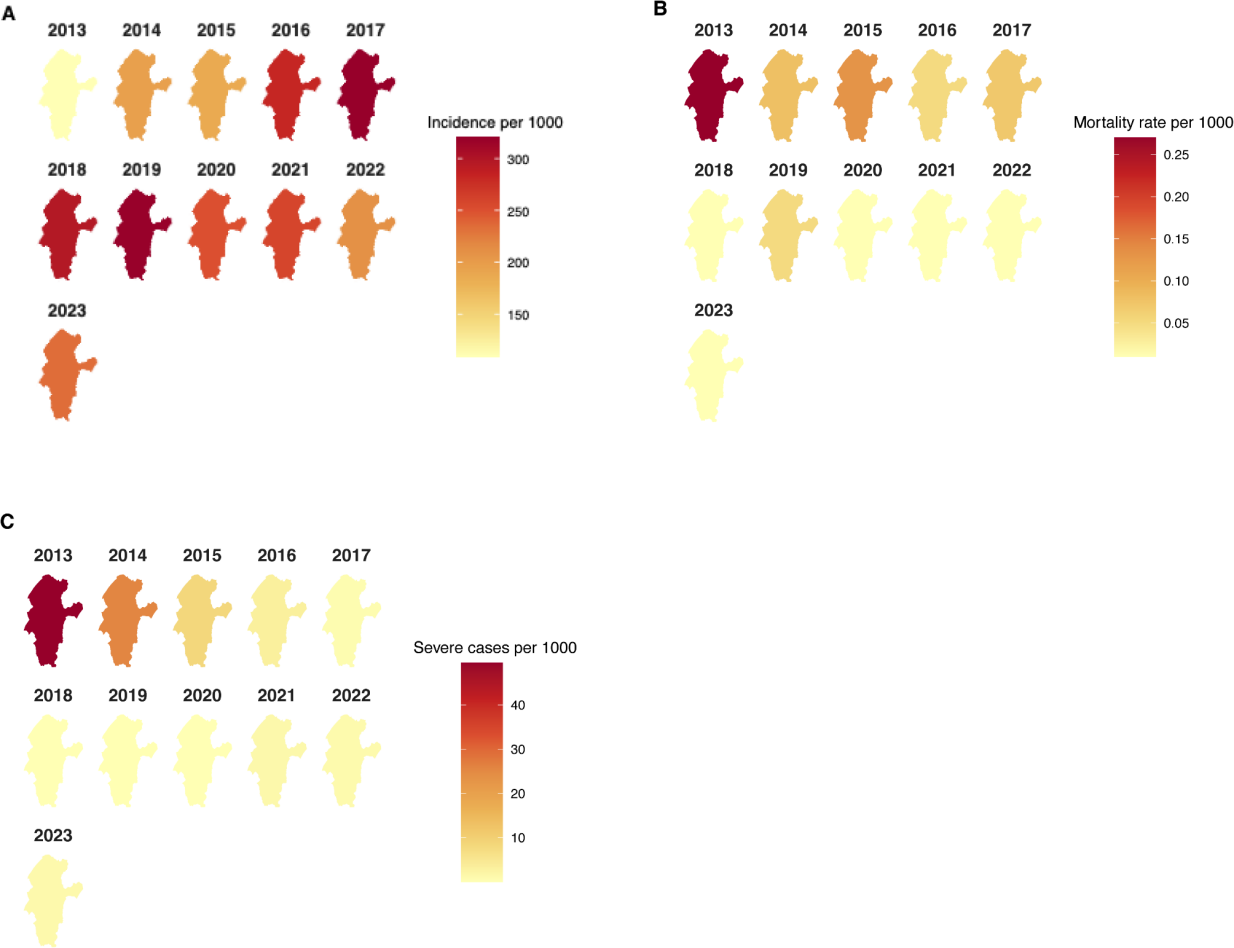
Malaria burden in Tarkwa-Nsuem district (2013–2023) **A**. Incidence rate **B**. Mortality rate **C**. Severe cases

Monthly data on malaria uncomplicated cases, severe cases, death cases, and population of children under five years between January 2013 and December 2023, were obtained from the Tarkwa- Nsuaem Municipal Health Directorate as displayed in Fig. 2. Data on the interventions were acquired from the Malaria Atlas Project (MAP) [20] sing the geographical coordinates of Tarkwa-Nsuaem. The Malaria Atlas Project is a global initiative that compiles spatial data about malaria transmission and intervention coverage.

- Insecticide-treated nets: Data on ITNs was obtained from MAP, which included ITNs distributed annually and usage recorded by households with children under five. Data on annual ITN distribution and household usage rates were extracted from MAP. ITNs were distributed through mass campaigns, antenatal care visits, and community-based programs. Coverage was calculated as the proportion of households with at least one ITN over the total number of households surveyed annually. Usage rates were assessed using household survey data, which recorded the proportion of children under five reported to have slept under an ITN the night before the survey.
- Treatment: The MAP provided information on ACT treatment regimens, specifically the use and availability of antimalarial medications and treatments. This was about distribution and access to therapy within the municipality. Information on treatment regimens focused on ACT, which is the first-line treatment for uncomplicated malaria in Ghana. Coverage was calculated as the proportion of confirmed malaria cases that received ACTs, based on health facility records. Timeframes for data collection were annual, reflecting updates from facility reporting systems.

Meteorological data such as temperature and rainfall, was collected from the Global Climate Monitor (https://www.globalclimatemonitor.org/) website using the geographical coordinates of Tarkwa-Nsuaem. Temperature data provided monthly average temperature measurements aggregated annually to represent them. Rainfall data was also acquired through monthly measurements, which are then summarized to provide yearly rainfall estimates.

### Data analysis

A yearly incidence rate was calculated by dividing the number of reported malaria cases by the population size from 2013 to 2023 (expressed as cases per 1,000 population). The mortality rate was determined by dividing the number of malaria-related deaths by the population size during the same period (expressed as deaths per 1,000 population). The case fatality ratio (CFR) represented the proportion of deaths among malaria cases, calculated as deaths per 1,000 cases. For the mean incidence and mortality rates, the denominators were the total population size across all years of the study, and the numerators were the cumulative number of malaria cases and deaths, respectively. Categorical variables were presented as frequencies (n) and percentages (%). Seasonal malaria trend from 2013 to 2023 was investigated using monthly data from the DHIMS-2 system for Tarkwa-Nsuaem, while the remaining analyses utilized data from the Statistical Yearbook. The Mann-Kendall test was applied to identify annual malaria trends. Python was used for data extraction, cleaning, and analysis. The geographical distribution of malaria was visualized with thematic maps created in R programming. Statistical significance for two-tailed P-values was set at α < 0.05.

Joinpoint regression is an effective tool for detecting changes in trends over time [21], while interrupted time series analysis is commonly used to evaluate the impact of a single intervention over a short period [22]. This study focused on analyzing the changes in malaria incidence and mortality from 2013 to 2023, aiming to understand the influence of multiple malaria interventions implemented either concurrently or consecutively. The annual percentage change (APC) was estimated using incidence data, as population changes in Tarkwa-Nsuem during this period made total case counts less reliable.

The analysis used the permutation test, assessed by the Monte Carlo method, to determine the number of significant joinpoints. The segmented regression function was fitted using a grid search method (GSM), allowing for up to two segments (one joinpoint) in the models. Joinpoint regression, with year as the regression variable, estimated the APC along with 95% confidence intervals (CI) between change points. The Z-test was applied to test whether the APC differed significantly from zero. Significant APC values (*P* < 0.05) indicated an increasing or decreasing trend, while non-significant values suggested a stable incidence. All joinpoint regression analyses were conducted using the Joinpoint Regression Program (version 4.8.0.1, National Cancer Institute, MD, USA).

To model the relationship of malaria incidence while accounting for external environmental influences, we employed the AutoRegressive Integrated Moving Average with Exogenous Variables (ARIMAX) model. ARIMAX extends the traditional AutoRegressive Integrated Moving Average (ARIMA) model by incorporating independent variables (exogenous predictors) that may influence the dependent variable over time. In this study, temperature and rainfall were used as exogenous variables to assess their impact on malaria incidence. The ARIMAX model is represented as ARIMAX(p, d, q), where p is the Autoregressive order, representing the number of past malaria incidence values used to predict future values; d is the Differencing order, indicating the number of times the data is differenced to remove trends and achieve stationarity; and q is the Moving Average order, referring to the number of past forecast errors used to improve predictions. X represents additional independent variables such as temperature and rainfall that influence malaria incidence. Since malaria incidence exhibits seasonal patterns, we extended the ARIMAX model to Seasonal AutoRegressive Integrated Moving Average with eXogenous variables (SARIMAX), denoted as $\:SARIMAX(p,\:d,\:q)\:\times\:\:(P,\:D,\:Q,\:s),$ where P, D, and Q represent the seasonal components of AR, I, and Moving Average, respectively, and s = 12 months to capture yearly fluctuations in malaria incidence. For this study, the best-fitting model was determined to be SARIMAX(1,1,1)x(1,1,1,12) based on Akaike Information Criterion (AIC) and Bayesian Information Criterion (BIC) values.

To ensure the robustness of the model, the Augmented Dickey-Fuller (ADF) test was conducted to check for stationarity, and first-order differencing was applied where necessary to remove trends. The Autocorrelation Function (ACF) and Partial Autocorrelation Function (PACF) plots were analyzed to determine the optimal values for p, d, q, and seasonal components (P, D, Q, s). Temperature and Rainfall were selected as exogenous variables due to their biological relevance to malaria transmission. The Ljung-Box test was conducted to check for autocorrelation in residuals, and the Jarque-Bera test was used to assess the normality of residuals.

## Results

### Descriptive statistics

Table 1 displays the descriptive statistics for monthly malaria burden data. It shows the various minimum, maximum, mean and standard deviation of the monthly data from 2013 to 2023. It is observed that the incidence cases of malaria remain high with approximately 1427 cases recorded with about 243 severe and 4 deaths between 2013 and 2023.

**Table 1:** Descriptive statistics of monthly cases, deaths, temperature and rainfall from 2013–2023

Variables	Minimum	Maximum	Mean	Standard Deviation
Cases	166	1427	838.91	270.61
Severe Cases	0	243	25.91	50.30
Death	0	4	0.19	0.64
Temperature	25.0	30.3	27.73	1.28
Rainfall	0.7	603.5	144.12	100.43

### Time series analysis of incidence cases, severe cases, and mortality cases

**Fig. 3:**
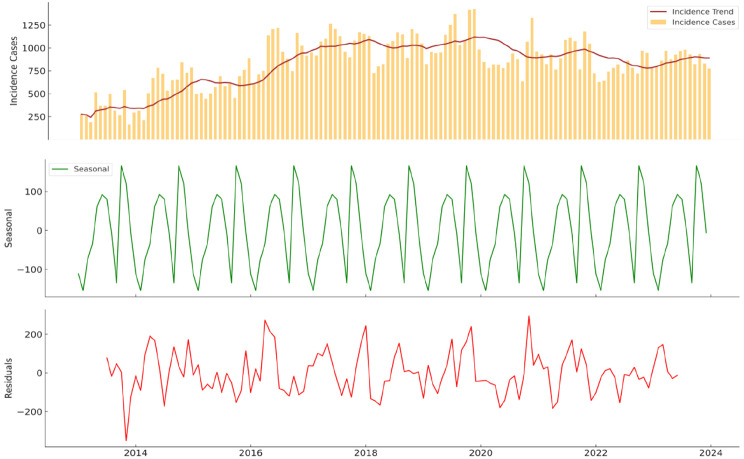
Trend, seasonal and Residual decomposition of malaria cases

From 2013 to 2023, a total of 110 737 malaria cases were reported, with an annual average incidence rate of 244.24 cases per 1,000 people. In total, 25 deaths were recorded, yielding an annual mean mortality rate of 0.059 deaths per 1,000 population and a CFR of 0.37 deaths per 1,000 cases. During the study period, the incidence rate rose by 12.48%, from 109.63 cases per 1,000 population in 2013 to 234.41 cases per 1,000 population in 2023 (Mann-Kendall test, *P* = 0.02). The mortality rate followed a downward trend with minor deviations. Between 2013 and 2023, the CFR reduced by 100%, from 2.46 to 0 deaths per 1000 patients. (as shown in Figs. 2 and 3).

Malaria in Tarkwa-Nsuaem had a perennial pattern, with the incidence rate peaking twice in a single year, as shown in Fig. 3. The incidence trend appeared to be cyclical, as the trend did not drastically change over time in terms of overall volume. Every year, it peaks between April and July, followed by October and December. This clearly showed the perennial nature of malaria transmission in the Tarkwa-Nsuem district. The Seasonal component presented regular oscillations, with values rising and falling in a consistent annual pattern. Its effect accounted for approximately +/- 50 cases per month from the baseline. The Residual component, which showed irregularities, generally fluctuates around zero with occasional spikes. The residual values also showed deviations from the predicted trend and seasonality, which could range around +/- 100 cases.

**Fig. 4:**
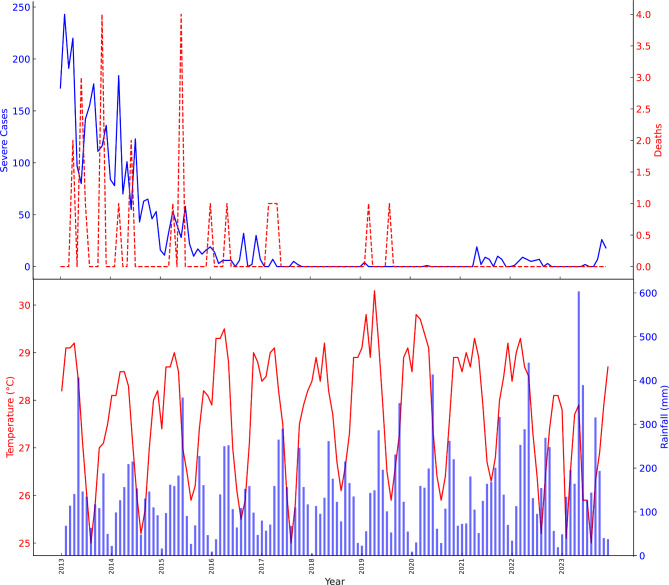
Trend of Severe Cases, Death, Temperature and Rainfall patterns in the Tarkwa-Nsuaem Municipality

The temperature in Tarkwa-Nsueam was consistently high throughout the year, as seen in Fig. 4. Interannual variations were evident in the rainfall, which was primarily focused during the summer months of June through September when malaria prevalence peaked. Temperature remains consistently high with minor fluctuations, typically between 28 °C and 30 °C. Rainfall displays significant peaks that are highly variable, ranging from as low as 0.7 mm to over 400 mm.

## Impact of rainfall and temperature on malaria incidence

Tables (2, 3 and 4) displays results of the ARIMAX model used to analyse the relationship between malaria incidence and exogenous environmental factors, specifically temperature and rainfall. The model incorporates autoregressive, moving average, and seasonal components while considering external predictors to determine their impact on malaria cases over time. The model specified was SARIMAX with malaria incidence cases as the dependent variable and temperature and rainfall as exogenous variables, accounting for a 12-month seasonal lag.

The results indicate that rainfall is a significant predictor of malaria incidence, with a coefficient of 0.2954 and a p-value of 0.032, making it statistically significant at the 5% level. Time-series dependencies were observed, with AR(1) at -0.7567 (*p* < 0.001), showing that past malaria cases significantly influence future cases. The seasonal moving average component (MA(12) = -0.5119, *p* < 0.001) confirms that malaria incidence follows a seasonal pattern, where previous year’s cases impact current trends. Model fit to determine the suitability of the SARIMAX(1, 1, 1)x(1, 1, 1, 12) was assessed using AIC (1336.92) and BIC (1355.50), with residual tests indicating no strong autocorrelation and no significant skewness.

**Table 2:** Model summary of ARIMAX model

Parameter	Estimates
Dependent Variable	Malaria Incidence Cases
No. of Observations	132
Model Type	SARIMAX(1, 1, 1)x(1, 1, 1, 12)
Log Likelihood	-661.462
AIC	1336.923
BIC	1355.501
HQIC	1344.451

**Table 3:** Variable estimates of ARIMAX model

Variable	Coefficient	standard error	Z	*P*>|z|	[0.025	0.975]
Temperature	$\:-2.7415$	$\:30.035$	$\:-0.091$	$\:0.927$	$\:-61.608$	$\:56.125$
Rainfall	$\:0.2954$	$\:0.138$	$\:2.145$	$\:0.032$	$\:0.025$	$\:0.565$
ar.L1	$\:-0.7567$	$\:0.212$	$\:-3.561$	$\:0.000$	$\:-1.173$	$\:-0.340$
ma.L1	$\:0.5854$	$\:0.270$	$\:2.168$	$\:0.030$	$\:0.056$	$\:1.114$
ar.S.L12	$\:-0.1784$	$\:0.137$	$\:-1.303$	$\:0.193$	$\:-0.447$	$\:0.090$
ma.S.L12	$\:-0.5119$	$\:0.134$	$\:-3.815$	$\:0.000$	$\:-0.775$	$\:-0.249$
sigma2	$\:1.684\:\times\:\:{10}^{4}$	$\:603.5$	$\:144.12$	$\:100.43$	$\:1.21\:\times\:\:{10}^{4}$	$\:2.16\:\times\:\:{10}^{4}$

**Table 4:** Statistical tests of ARIMAX model

Test	Estimate
Ljung-Box (L1)	0.81
Prob(Q)	0.37
Jarque-Bera (JB)	1.54
Prob(JB)	0.46
Heteroskedasticity (H)	0.47
Skew	-0.29
Prob(H) (two-sided)	0.03
Kurtosis	3.08

### Joinpoint analysis of incidence rate, severe cases rate, and mortality rate

Incidence Rate showed a significant increase in observed rates from 2013 to 2017 as seen in Fig. 4. After 2017, there was a sharp decrease and the joinpoint indicates a major change was observed from 2017 to 2023. The Average Annual Percent Change (AAPC) for the entire period from 2013 to 2023 is 5.7266% with a 95% confidence interval ranging from − 0.2147 to 12.0217%. However, this change was not statistically significant, as indicated by a p-value of 0.059143. This shows different patterns in the two segments. Between 2013 and 2017, there was a significant increase in malaria incidences over time with an Annual Percent Change (APC) of 28.1171% (95%CI:11.2868%, 47.4928%; p-value = 0.005065). The cases have been rising since, with malaria notified across the state. The trend changed between 2017 and 2023, with an APC of -6.9815% (95% Confidence Interval: -13.7268–0.2911%) However, this decrease was not statistically significant at 0.05 level (*p* = 0.56871).

**Fig. 5:**
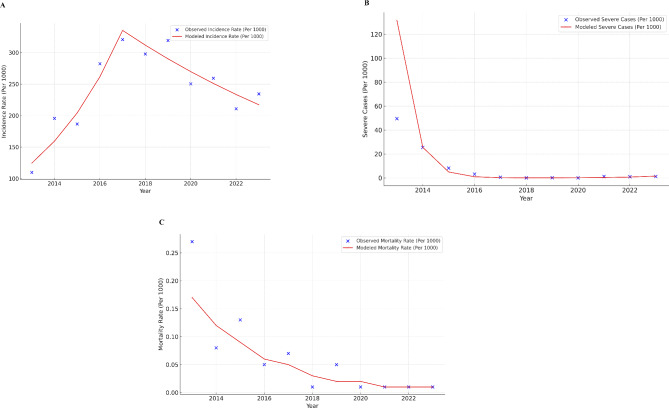
Trend of (**A**) Incidence rates per 1000 (under 5 ages); (**B**) Severe rates per 1000 (under 5 age); (**C**) Mortality rates per 1000 (under 5 ages); of malaria in Tarkwa-Nsuaem of Ghana between 2013 and 2023

Between 2013 and 2018, there was a significant annual decrease of 80.6% in severe malaria cases (*p* = 0.002381) as shown in seen in Fig. 5B. Between 2018 and 2023, there was a yearly increase of 110.3%, however, this growth was not considered statistically significant (*p* = 0.062835). On average annually, there was a 36.1% decrease in severe cases (Per 1000) of malaria under 5 from 2013 to 2023. This general trend was not statistically significant at the 0.05 level (*p* = 0.051806) likely because of the change of direction in the trend after 2018. The APC from 2013 to 2023 amounts to a statistically significant − 27.91% per year (*p* < 0.05), indicating a notable and consistent reduction in the malaria death rate over this timeframe as seen in Fig. 5C. The AAPC for the same period also reflects a yearly decline of -27.91%, consistent with the APC. (as seen in Table 5)

**Table 5:** Annual percentage change (APC) of incidence rate, severe cases (Per 1000), and mortality rate

Indicator	Segment	Years	APC^a^ (95% CI)	T^b^ (*P*-value)
Incidence Rate	1	2013–2017	28.12 (11.29–47.49)	4.31 (0.005)
	2	2017–2023	-6.98 (13.73 − 0.29)	-2.35 (0.057)
	All	2013–2023	5.73 (-021-12.02)	1.89 (0.059)
Severe Cases (Per 1000)	1	2013–2018	-80.60 (91.26; -56.92)	-5.03 (0.002)
	2	2018–2023	110 (-5.3; 366.94)	2.28 (0.063)
	All	2013–2023	-36.13 (-59.35; 0.35)	-1.95 (0.052)
Mortality Rate	All	2013–2023	-27.91 (-36.76; -17.82)	-5.65 (< 0.001)

^a^ Annual Percent Change; ^b^ Test statistic

### Malaria intervention implementation and changes in Tarkwa-Nsuaem

**Fig. 6:**
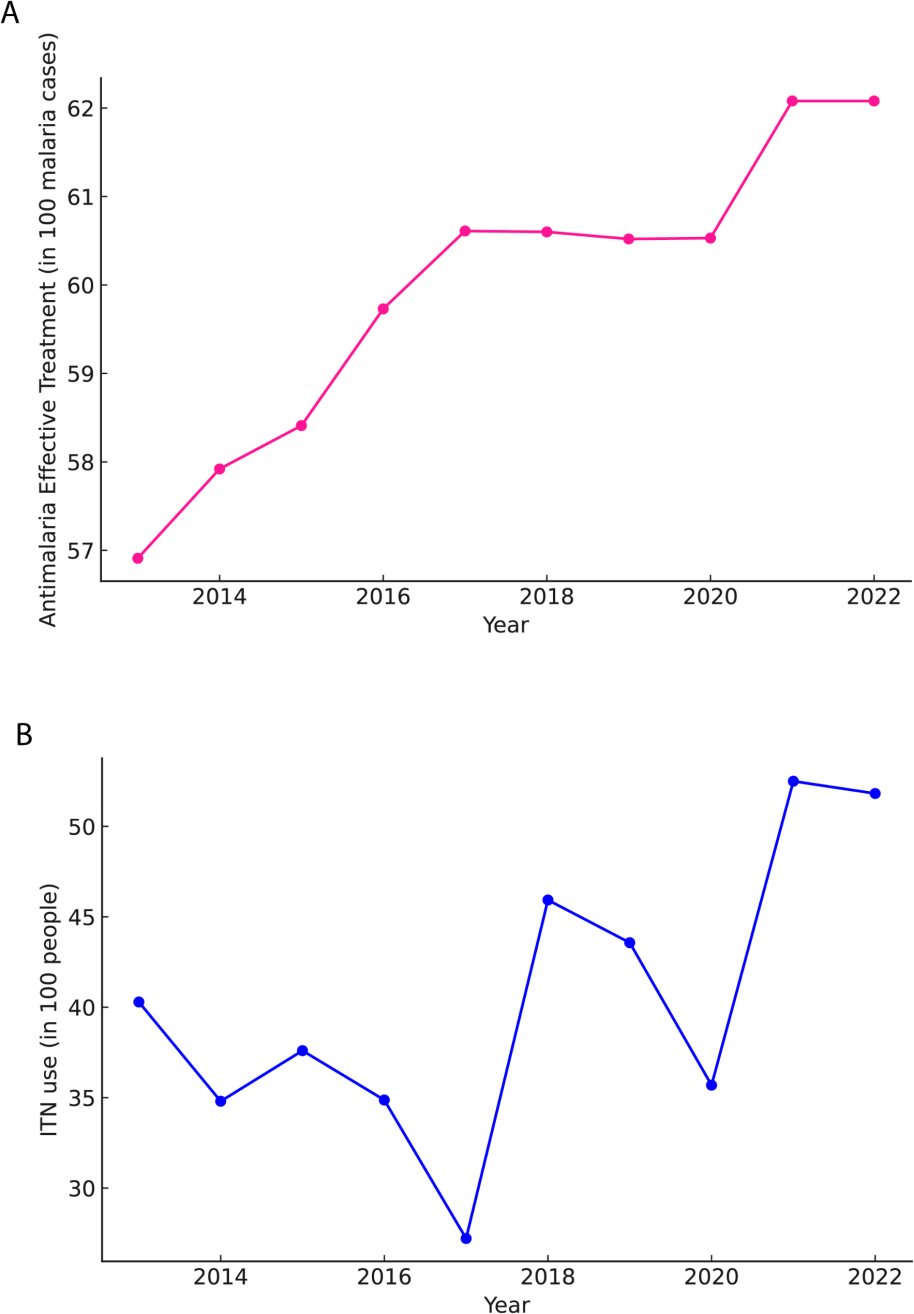
**Trends of intervention coverage for the Tarkwa Nsuaem Municipality (A) Case management (B) ITN usage**

The data on interventions from MAP implemented in Tarkwa-Nsuaem shows distinct trends from 2013 to 2023 (Fig. 6). With malaria cases data, in 2013, the municipality recorded 4,074 malaria under 5 cases, with an ITN coverage of 40.29% and a treatment coverage of 56.91%. Ten malaria-related deaths occurred that year. By 2016, the number of cases had increased to 11,340, while ITN coverage had decreased to 34.87%. Treatment coverage had risen to 59.73%, and the number of deaths had decreased to two. The years 2017–2019 saw the highest recorded malaria cases in the municipality. Cases peaked at 13,387 in 2019. ITN coverage during this period fluctuated, dropping to its lowest point of 27.21% in 2017 before rising to 43.57% in 2019. Treatment coverage remained relatively stable, ranging between 60.52% and 60.61%.

From 2020 to 2023, the annual case numbers were lower than the 2019 peak. The municipality recorded 10,713 cases in 2020, 11,334 in 2021, 9,414 in 2022, and 10,689 in 2023. ITN coverage reached its highest levels during this period, rising to 52.5% in 2021 and maintaining around 51.82% through 2023. Treatment coverage also reached its highest level at 62.08% from 2021 onwards. Notably, no malaria-related deaths have been recorded in the municipality since 2020. The population of Tarkw-Nsuaem grew from 37,160 in 2013 to 45,599 in 2023. The malaria incidence rate per 1,000 population varied over this period: 109.63 in 2013, reaching a peak of 320.69 in 2017 and standing at 234.41 in 2023. Throughout the decade, treatment coverage showed a consistent upward trend, starting at 56.91% in 2013 and reaching 62.08% by 2021, where it has remained. ITN coverage, however, fluctuated significantly: starting at 40.29% in 2013, dropping to 27.21% in 2017, and then rising to maintain levels above 50% from 2021 to 2023.

In 2023, Tarkwa-Nsuaem, a district with a population of 45,599, reported an annual malaria case count of 10,689. The district achieved an insecticide-treated net (ITN) coverage of 51.82% and treatment coverage of 62.08%. Remarkably, there were zero malaria-related deaths for the fourth consecutive year, highlighting effective healthcare interventions and management strategies. The malaria incidence rate stood at 234.41 cases per 1,000 population, reflecting ongoing efforts to control and reduce malaria transmission in the district.

## Discussions

This study provides a comprehensive analysis of malaria trends in Tarkwa-Nsuaem Municipality over the past decade, highlighting both successes and persistent challenges in malaria control efforts. The findings revealed an overall increase in malaria incidence between 2013 and 2023, despite national and local intervention programs. Malaria-related deaths were eliminated from 2020 to 2023, marking a significant public health achievement. A key finding of this study is the seasonal pattern of malaria transmission, with cases peaking during the rainy seasons (April–July and October–December). The ARIMAX model confirms that rainfall plays a critical role in malaria incidence, while temperature does not have a direct impact. The model successfully captures seasonal trends, reinforcing that malaria cases are influenced by historical trends and rainfall patterns. This pattern confirms the strong link between rainfall and malaria incidence, as has been observed in previous studies in Ghana and other malaria-endemic regions of Africa [23, 24, 25].

In this study, the incidence of malaria in the Tarkwa-Nsuaem district was not significantly influenced by temperature. A similar observation was made in the Wassa Amenfi West district of the Western Region of Ghana, where the incidence of malaria was found to be negatively correlated with temperature [25]. Furthermore, an estimation study conducted in Ghana on the influence of temperature and rainfall on malaria incidence indicated that temperature had a statistically significant negative impact on malaria, while rainfall, with a delay of several months, had a statistically significant positive impact [26]. The results of the present study in the context of Tarkwa-Nsuaem are consistent with these findings.

A negative correlation between temperature and malaria incidence has been reported in West Africa in additional studies [27], while an insignificant correlation was observed in certain regions of Africa, particularly Ethiopia [28]. The complex nature of malaria epidemiology is further highlighted by these variations, which emphasises the necessity of a comprehensive investigation that employs a variety of methods. It is also important to recognise that malaria incidence is not solely determined by meteorological factors. Transmission patterns are significantly influenced by a variety of factors, including humidity, population movement, economic activities, and the utilisation of malaria interventions.

A significant challenge identified in this study is the fluctuation in ITN coverage over time and the lack of monthly interventional data. ITN distribution declined from the period of 2013 to 2017, before recovering in 2023. The inconsistency in vector control may have contributed to the observed increase in malaria cases, particularly between 2013 and 2019, when cases peaked in 2019. Studies from other mining communities in Ghana report similar difficulties in maintaining consistent ITN coverage, suggesting that standard distribution models may not be sufficient for areas with high population movement and environmental disruptions [29, 30]. The significant expansion of malaria treatment coverage, which experienced exponential growth from 2013 to 2023, likely contributed to the reduction in severe malaria cases and the elimination of malaria-related deaths. The complete elimination of malaria mortality since 2020 aligns with Ghana’s national malaria control objectives, emphasizing the importance of malaria interventions such as ITN distribution and effective case management together with timely access to treatment [31].

Mining activities can create additional breeding sites for mosquitoes and affect local ecology in ways that impact malaria transmission [8, 16]. This creates mosquito breeding sites through stagnant water accumulation, altering local ecology and increasing malaria transmission as indicated in other setting facing similar mining challenges [8]. The municipality’s growing population, which increased in 2023 according to the Ghana Statistical Service [19], may have strained healthcare resources and complicated intervention coverage, a challenge observed in other rapidly growing communities in Ghana [8]. Despite these challenges, the success in eliminating mortality in 2018 and from 2020 to 2023 while facing increasing case numbers suggests effective interventional strategies.

## Limitations

The study draws attention to the need for further studies on entomology, intervention coverage and impacts. It also underscores the need for additional investments in the collection of subnational surveillance data. Evidence-based and effectively targeted national programs require high-quality data. Obtaining district-level intervention coverage data specifically, monthly data on intervention coverages for ITNs and case management were unavailable on a yearly basis. This lack of granular data made it challenging to conduct a thorough analysis of the causal effect of these interventions on malaria incidence. Furthermore, because routine data frequently lacks the resolution required to precisely capture changes in intervention impact and transmission patterns across different regions, using it could be misleading.

Variations in population migration, the behaviour of local vectors, and environmental factors that affect the transmission of malaria may not always be taken into account by routine data sources. Inconsistent data reporting and data quality might make it more difficult to evaluate the results and possibly distort our perception of how effective an intervention is. To create a complete and more accurate picture of malaria dynamics at the subnational level, future initiatives should concentrate on improving data collection techniques and combining diverse data sources.

## Conclusion

The most recent years show a declining trend in both absolute case numbers and incidence rates, suggesting that interventions are successfully outpacing population growth. This achievement, combined with the elimination of malaria deaths since 2020, indicates that while challenges remain, the municipality is making substantial progress in its implementation of national malaria control strategies. Tarkwa-Nsuaem continues to face challenges in fully implementing national strategies. Tarkw-Nsuaem’s malaria control efforts must continue to align with and build upon national strategies. Future directions should focus on further increasing ITN coverage and usage and expanding treatment coverage. Future malaria control efforts in Tarkwa-Nsuaem should focus on addressing the bimodal seasonal peaks through targeted interventions, potentially including enhanced vector control during high-transmission periods. Additionally, the impact of mining activities on malaria transmission patterns should be studied to develop more effective control measures.

## Data Availability

The secondary data on malaria incidence that supports the findings of this study are available from the authors with permission from the Western Regional Health Directorate through the District Health Information Management System (DHIMS) and so are not publicly available. Data on coverages of intervention was assessed from Malaria Atlas Project website and are available at the following URL: https://data.malariaatlas.org.

## Abbreviations

ITN: Insecticide-Treated Net
ACT: Artemisinin-based Combination Therapy
IPTp: Intermittent Preventive Treatment in Pregnancy
SMC: Seasonal Malaria Chemoprevention
NMCP: National Malaria Control Programme
RBM: Roll Back Malaria
IRS: Indoor Residual Spraying
RTS, S: Malaria vaccine (RTS, S/AS01)
WHO: World Health Organization
CFR: Case Fatality Ratio
ARIMAX: Autoregressive Integrated Moving Average with Exogenous Variables
ARIMA: Autoregressive Integrated Moving Average
ADF: Augmented Dickey-Fuller
SARIMAX: Seasonal Autoregressive Integrated Moving Average with Exogenous Variables
ACF: Autocorrelation Function
PACF: Partial Autocorrelation Function
